# Supplementary material for: Cross-Linking Reaction of Bio-Based Epoxy Systems: An Investigation into Cure Kinetics
Source: Polymers (Basel). 2024 Sep 2;16(17):2499. doi: 10.3390/polym16172499 (PMC11397831; doi:10.3390/polym16172499)
Supplement: Supplementary file 1 [file polymers-16-02499-s001.zip › polymers-3134420-supplementary.pdf]

# Supplementary Material

## S1. Glass transition temperature

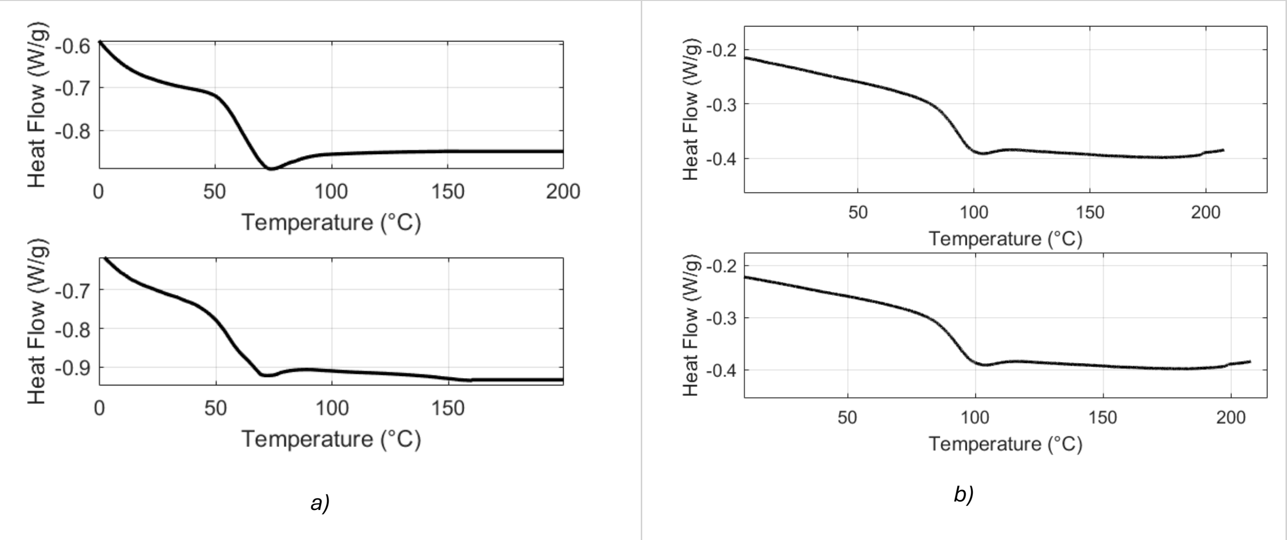


**Figure S1.** DSC curves vs temperature on samples after curing: (a) M1 and M1-20% NC547, (b) M2 and M2-20% NC514 (exo-up)

## S2. Activation Energy

**Table S1.** Activation energy values

| Sample | Ea* (kJ/mol)** | Ea max (kJ/mol)** | Ea min (kJ/mol)** |
|---|---|---|---|
| M1 | 47.8 | 57.2 | 40.8 |
| M1+20% NC547 | 47.2 | 50.5 | 45.2 |
| M2 | 57.2 | 67.8 | 54.8 |
| M2+20% NC514 | 49.7 | 55.4 | 44.3 |

* Calculated as average value

** These values refer to the conversion range between 0.1 and 0.9

## S3. Kamal Sourour fit

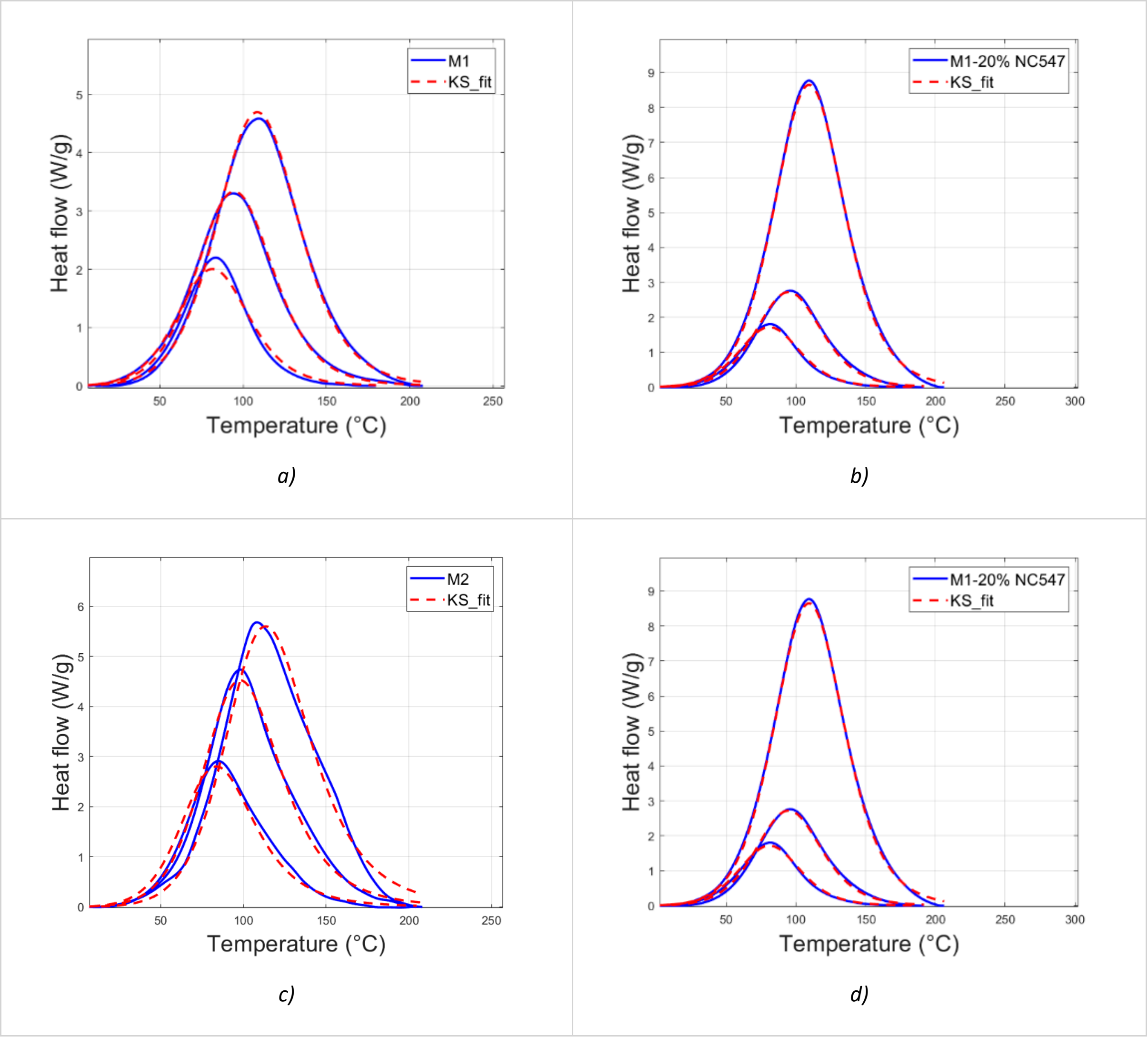


**Figure S2.** Heat flow vs temperature graphs (both experimental and simulated) for M1 (a), M1 + 20%NC547 (b), M2 (c), and M2 + 20% NC514 (d)
